# Deep-brain stimulation of the human nucleus accumbens-medial septum enhances memory formation

**DOI:** 10.21203/rs.3.rs-3476665/v1

**Published:** 2023-11-20

**Authors:** Svenja Treu, Juan A Barcia, Cristina Torres, Anne Bierbrauer, Javier J. Gonzalez-Rosa, Cristina Nombela, Jose A Pineda-Pardo, Daniel Torres, Lukas Kunz, Robin Hellerstedt, Josue M Avecillas-Chasin, Monica Lara, Marta Navas, Ana Galarza Vallejo, Julia García-Albea, Antonio Oliviero, Fernando Seijo, Andreas Horn, Ningfei Li, Nikolai Axmacher, Santiago Canals, Blanca Reneses, Bryan A Strange

**Affiliations:** 1 Laboratory for Clinical Neuroscience, Centre for Biomedical Technology, Universidad Politécnica de Madrid, Spain; 2 Department of Neurosurgery, Hospital Clínico San Carlos, Instituto de Investigación Sanitaria San Carlos, Universidad Complutense de Madrid, Spain; 3 Department of Neurosurgery, University Hospital La Princesa, Madrid, Spain; 4 Institute of Systems Neuroscience, Center for Experimental Medicine, University Medical Center Hamburg-Eppendorf (UKE), Germany; 5 Departament of Psychology, University of Cadiz, Institute of Biomedical Research Cadiz (INiBICA), Cádiz, Spain.; 6 Departamento de Psicología Biológica y de la Salud, Facultad de Psicología, Universidad Autónoma de Madrid, Madrid, Spain; 7 HM CINAC (Centro Integral de Neurociencias Abarca Campal), Hospital Universitario HM Puerta del Sur, HM Hospitales, Madrid, Spain; 8 Instituto de Neurociencias, Consejo Superior de Investigaciones Científicas & Universidad Miguel Hernández, Sant Joan d’Alacant, Spain; 9 Department of Biomedical Engineering, Columbia University, New York, NY, USA; 10 Department of Neurosurgery, University of Nebraska Medical Center, Omaha, NE, USA; 11 Department of Neurosurgery, University of California Los Angeles, Los Angeles, CA, USA; 12 Department of Neurosurgery, Hospital Universitario Fundación Jiménez Díaz, Madrid, Spain; 13 Department of Psychiatry, Hospital Clínico San Carlos (IdISSC), CIBERSAM, Universidad Complutense de Madrid, Spain; 14 Hospital Nacional de Parapléjicos, FENNSI Group, Toledo, Spain; 15 Centro Medico Asturias, Oviedo, Spain; 16 Movement Disorder and Neuromodulation Unit, Department of Neurology, Charité – Universitätsmedizin Berlin, corporate member of Freie Universität Berlin and Humboldt-Universität zu Berlin, Department of Neurology, 10117 Berlin, Germany; 17 Center for Brain Circuit Therapeutics Department of Neurology Brigham & Women’s Hospital, Harvard Medical School, Boston MA 02115, USA; 18 MGH Neurosurgery & Center for Neurotechnology and Neurorecovery (CNTR) at MGH Neurology Massachusetts General Hospital, Harvard Medical School, Boston, MA 02114, USA; 19 Department of Neuropsychology, Institute of Cognitive Neuroscience, Faculty of Psychology, Ruhr University Bochum, Germany; 20 Department of Neuroimaging, Alzheimer’s Disease Research Centre, Reina Sofia-CIEN Foundation, Madrid, Spain

## Abstract

Deep-brain stimulation (DBS) is a potential novel treatment for memory dysfunction. Current attempts to enhance memory focus on stimulating human hippocampus or entorhinal cortex. However, an alternative strategy is to stimulate brain areas providing modulatory inputs to medial temporal memory-related structures, such as the nucleus accumbens (NAc), which is implicated in enhancing episodic memory encoding. Here, we show that NAc-DBS improves episodic and spatial memory in psychiatric patients. During stimulation, NAc-DBS increased the probability that infrequent (oddball) pictures would be subsequently recollected, relative to periods off stimulation. In a second experiment, NAc-DBS improved performance in a virtual path-integration task. An optimal electrode localization analysis revealed a locus spanning postero-medio-dorsal NAc and medial septum predictive of memory improvement across both tasks. Patient structural connectivity analyses, as well as NAc-DBS-evoked hemodynamic responses in a rat model, converge on a central role for NAc in a hippocampal-mesolimbic circuit regulating encoding into long-term memory. Thus, short-lived, phasic NAc electrical stimulation dynamically improved memory, establishing a critical on-line role for human NAc in episodic memory and providing an empirical basis for considering NAc-DBS in patients with loss of memory function.

The NAc is a small, targetable gateway with widely projected cognitive effects across the entire brain, including motivation and learning. Its key anatomical position linking mesolimbic dopaminergic and limbic structures, basal ganglia, mediodorsal thalamus and prefrontal cortex^1, 2^ has rendered this nucleus an attractive target for DBS treatment of medication-resistant psychiatric disorders^3^, and also implies that stimulating this structure may have far-reaching effects on cognition^4, 5^. While cognitive studies on NAc function have primarily focused on reward processing^6^ and reinforcement learning^7^, there is cross-species evidence from animal models^2^ and human neuroimaging studies^8–10^ for a role for NAc in upregulating episodic memory for salient events. This is thought to reflect the central position of this nucleus in a circuit linking the hippocampus, the brain structure critical for episodic memory^11^, to the dopaminergic ventral tegmental area (VTA)^2^. Beyond reward and salience, declarative memory formation in general has also been linked to the nucleus accumbens^12^ and learning impairments similar to those seen after hippocampal lesions have been observed as a result of NAc lesions in non-human primates^13^. Furthermore, long-term improvements in cognitive or memory scores have been recorded following DBS of the nucleus accumbens^14, 15^. Cholinergic projections from the medial septum and diagonal band of Broca, which are located directly medially and posteriorly to the NAc, have also been shown to upregulate hippocampal activity^16, 17^ and lesions to basal forebrain cholinergic nuclei are known to impair memory^18^.

Direct, long-term, deep brain stimulation of the NAc (NAc-DBS) is employed in the management of several treatment-resistant psychiatric disorders, including obsessive-compulsive disorder (OCD)^3^, major depressive disorder (MDD)^14^ and anorexia nervosa (AN)^19^. Still, relatively little is known about the cognitive effects following DBS to this structure and thus far, studies have focused on long-term changes of standard neuropsychological measures, which are influenced by practice and placebo effects. We hypothesized that transient NAc-DBS would modulate memory function in these patients in a controlled “ON-OFF” design and tested this hypothesis in two experiments (Fig. 1).

In experiment 1 (Exp 1), 8 OCD patients and one AN patient (Table 1, **Supplementary Table 1**) who underwent bilateral NAc electrode implantation (Fig. 2a, **Supplementary Fig. S1**) took part in a visual memory task up to 6 weeks after surgery, with the stimulator switched off during this interval. Given the role of NAc in processing reward and positive valence^6, 8^, as well as contextually salient stimuli^9, 10^, we presented patients with neutral, pleasant and infrequent (“oddball”) pictures (Fig. 2b). That is, presented images were salient either because of their positive valence, or their infrequent occurrence (perceptual oddballs; photographs of black and white objects, by contrast to all other stimuli which were color scenes). During encoding, images were presented in 6 ‘periods’ (all stimulus types presented per period), with the critical manipulation being the application of bipolar stimulation between two electrode contacts in NAc during periods 3 and 5 (Fig. 2c), using standard clinical settings (130 Hz, 3.5V, 60μs pulse width). One hour later, patients performed a surprise recognition memory test. All stimuli shown at encoding were presented, randomly intermixed with an equal number of new foil items. Patients were required to make a push-button response to indicate whether they had seen the picture before.

Given that memory enhancement for salient stimuli is thought to reflect engagement of a hippocampal-mesolimbic loop^2^, we hypothesized that hippocampal involvement would be critical to any NAc-DBS effects on memory. To specifically test hippocampal involvement, we employed a recognition task requiring “remember” (R), “know” (K) or “new” (N) decisions^20^. Remember responses indicated that the patient could consciously recollect elements of the study episode (considered hippocampal-dependent^21^). Know responses, thought to rely on anteromedial temporal cortex^22^ (but see ^23^), indicated a sense of familiarity with the picture without being able to recollect any contextual information about its previous occurrence. New responses indicated the stimulus was not presented at encoding. Memory performance on this task was compared to a separate group of 9 patients with severe OCD but not undergoing DBS treatment, *i.e.*, control OCD group (cOCD) (**Supplementary Tables 2–3**).

In Exp 2, we applied an adapted version of a virtual-reality based path-integration task^24^ in 11 OCD patients and one AN patient chronically treated with NAc-DBS (Table 1, Fig. 2a). The multifaceted nature of path-integration requires the ability to keep movement directions, movement speeds, and time periods in memory up to the point where the homing vector has to be computed ^25–27^. Exp 2 was designed to test path integration as specifically as possible (for a detailed description, see ^24^) and it is unlikely that the subject’s performance in this task is driven by cognitive processes other than path integration. Evidence from rodent^25, 26, 28^ and human studies^24, 27, 29^ implicate the hippocampus in path integration. Exp 1 and 2 were therefore designed to recruit different aspects of memory function, but to be convergent in their hippocampal dependence. A further 12 healthy control subjects, matched in gender and age, also completed this experiment. In this task, patients navigated through a virtual environment featuring a grass landscape. Similar to previously established path-integration tasks^30^, each trial consisted of four successive steps: navigation to a goal location (indicated by an empty basket), which the subjects were instructed to remember; navigation to a distractor location (indicated by a tree); navigation to a retrieval location (indicated by a tree with an apple); and returning to the goal location and “dropping” the apple into the now invisible basket via a button press (*“drop location”*). The study comprised two subtasks, so that in half of the trials a lighthouse served as a local landmark (landmark-supported path integration; LPI), whereas in the other half of the trials no supportive spatial cues were available and participants had to rely on pure path integration (PPI). This task was also divided into 6 periods with stimulation applied either during periods three and five (7 patients) or during periods four and six (5 patients). Spatial memory accuracy was quantified by the Euclidean distance between the drop location and the correct goal location, referred to as “drop error”. Post-operative electrode localizations were assessed to map memory improvement across the two studies to anatomical space (“sweetspot mapping”). In Exp 3, a further 3 patients performed the same task as in Exp 1, but this time stimulating the electrode contacts nearest to the memory sweetspot. Probabilistic tractography was applied to preoperative MRI scans to verify that the stimulation site lies within a hippocampal-VTA circuit. In a fourth experiment, local and distant effects of NAc-DBS on neuronal activity were assessed with rodent functional MRI. A summary of all experiments and analyses reported here is provided in Fig. 1.

## RESULTS

### NAc-DBS during encoding enhances subsequent recollection of visual stimuli

**Figure 2.**
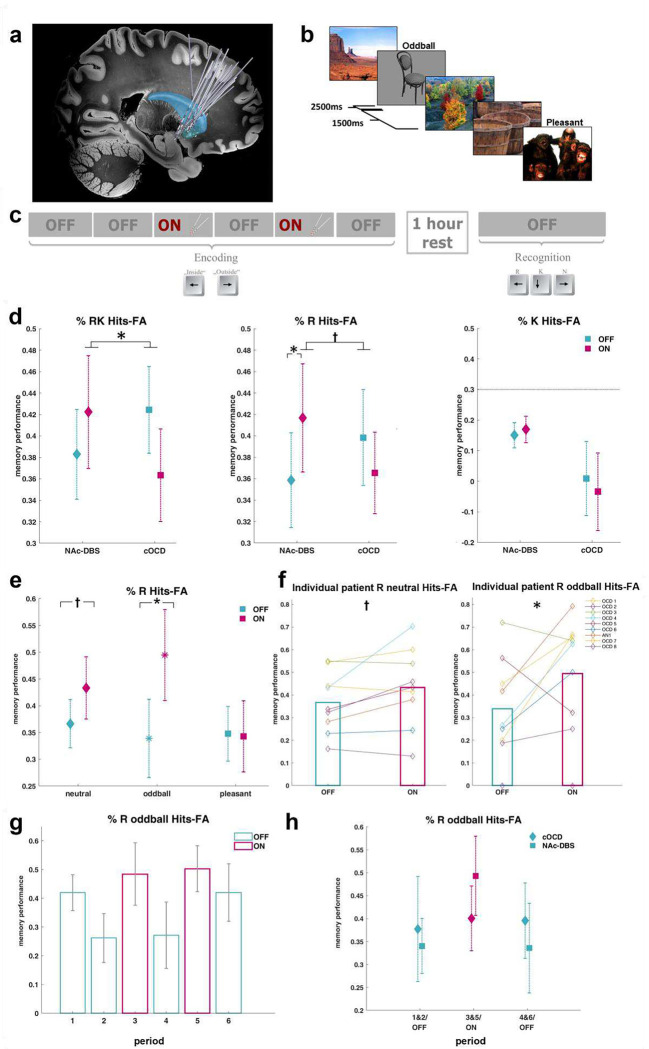
NAc-DBS enhances visual memory encoding of infrequent stimuli (Exp 1). **a.** Electrode positions of all patients participating in the memory tasks, with average patient-specific segmentations of caudate nucleus (blue) and NAc (green) and a 7 tesla ex vivo 100-micron T1 scan serving as background template (https://openneuro.org/datasets/ds002179/versions/1.1.0;). **b**. In each of 6 periods, emotionally neutral and positive stimuli, and infrequent oddball stimuli, were presented. **c**. The stimulator was set to ON during the 3^rd^ and 5^th^ periods, with stimulator settings manipulated in the ~10s interval in between periods. Each patient completed a surprise recognition test one hour after encoding. **d.** Relative to the OFF periods, encoding during NAc-DBS enhanced the probability that stimuli would later be correctly recognized. In the stimulated group (NAc-DBS), conscious recollection (R) for pictures presented during ON periods was significantly greater relative to OFF periods, while familiarity judgements (K) were not affected by DBS and no differences were found between the corresponding ON (3&5) and OFF (1,2,4&6) periods in the patient control group (cOCD). The dotted horizontal line in the plot of K performance (right panel) indicates the lower y-axis limit of the homologous plots for RK and R performance (left and middle panel). **e.** Significant DBS effect on the encoding of oddball stimuli, trend level significance for neutral pictures. **f**. Individual patient data is shown, superimposed on average memory scores across patients. **g.** Average scores of DBS patients for oddball memory accuracy are shown for each of the six periods separately**. h.** Data for both patient groups are presented for the three phases of the task: Baseline (period 1&2), ON (3&5), post-DBS (4&6). Error bars represent standard errors of the mean; * p<0.05; † p<0.1; FA: false alarms

In Exp 1, the probability of subsequently correctly recognizing stimuli was calculated separately for all stimulus types (neutral, pleasant and oddball) encoded during OFF (1, 2, 4 and 6) and ON (3 and 5) periods. This was done for both NAc-DBS and patient control groups (**Supplementary Data 1, Supplementary Tables 4–7**). “ON periods” in control patients refer to periods 3 and 5. We calculated correct remember and familiarity response rates, correcting for false alarm responses. For familiarity judgments, the independence assumption (familiarity = K/(1-R))^31^ was applied. In a first analysis, memory performance, calculated as correct hits-rate minus false alarm (FA), was pooled over R and K responses and compared between the two patient groups, for the three stimulus subtypes and the two DBS conditions. The group (DBS, control) by stimulation (ON, OFF) by subtype (neutral, pleasant, oddball) ANCOVA revealed a significant group by stimulation interaction (F_1,15_=5.5; p=0.033; η_p_^2^=0.27), with higher subsequent memory for pictures presented during NAc-DBS ON *vs*. OFF periods in DBS patients (Fig. 2d). Over all DBS patients, the mean (±sem) relative improvement in memory performance, calculated as (memory performance ON – memory performance OFF)/memory performance OFF, was 11.71 (6.93)% collapsing over stimulus subtypes. Given our hypothesis that NAc stimulation would modulate hippocampal function, and the reliance of remember judgments on the hippocampus, the analysis was then performed separately for the recognition response types. While familiarity judgements were not affected by DBS during encoding (group by stimulation K: F_1,15_=0.041; p=0.843; η_p_^2^=0.003), DBS patients, in comparison to cOCD patients, remembered more pictures encoded during ON *vs*. OFF periods, which reached trend level significance (group by stimulation R: F_1,15_=4.2; p=0.058; η_p_^2^=0.22). An analysis of the stimulated group only showed that NAc-DBS significantly improved encoding (stimulation R: F_1,7_=6.3; p=0.040; η_p_^2^=0.47) and interacted with stimulus type (stimulation by subtype R: F_2,14_=4.05; p=0.041; η_p_^2^=0.37; Fig. 2e–g, **Supplementary Fig. S2**). That is, simple main effects indicated that DBS significantly improved subsequent recollection of oddball stimuli (ON-OFF=17.49 ±sem 7.49%, relative improvement 72.14 ±sem 29.61%; p=0.030; 95% CI of ON-OFF difference = 2.3 to 32.7%), improved recollection of neutral stimuli at trend level significance (ON-OFF=7.10 ±sem 3.08% , relative improvement 18.28 ±sem 8.26%; p=0.066; 95% CI of ON-OFF difference = −0.6 to 14.8%) and had no effect on the encoding of pleasant stimuli (p=0.883). Recollection of oddball stimuli was enhanced by DBS in 7 out 9 patients. In control patients (not undergoing DBS), on the other hand, recognition accuracy did not differ between the periods of encoding corresponding to stimulation ON (3 & 5) vs. OFF (1, 2, 4 & 6) (p=0.725).

### Retrograde or anterograde effects of NAc-DBS

The mesolimbic dopamine system is thought to modulate memory retroactively, by influencing consolidation of memories after encoding^32^, as well as proactively^33^ which raises the possibility that stimulating the NAc could have influenced consolidation of preceding or succeeding periods. Only two periods, 2 and 6, could be used to analyze retrograde or anterograde effects, retrospectively, since the fourth period both preceded and succeeded DBS. Both retrograde and anterograde effects of DBS seemed unlikely here, because no significant differences between the DBS and patient control groups were found in period 2 (t_16_=0.89; p=0.390), or in period 6 (t_16_=0.06; p=0.953), for oddball stimuli R hits-FA. To examine a possible carry-over effect of DBS, periods 1 and 2 were defined as baseline, periods 3 and 5 as ON and periods 4 and 6 as post-DBS and, analyzing R hits-FA for oddball pictures, a significant quadratic effect was observed (F_1,7_=6.9; p=0.033; η_p_^2^=0.49; Fig. 2h) which confirmed that encoding success peaked during ON periods, highlighting a critical on-line role of this structure for memory enhancement. Given a possibility of an effect of DBS on subsequent response tendencies, we further analyzed the overall false alarm rates of R and K responses during the subsequent recognition task and observed no significant differences between the two patient groups (t_16_=0.04; p=0.971).

### Stimulation history modulates speed of responding at recognition

Reaction times (RTs) for the encoding task (indoor/outdoor judgments made by button press) were not significantly affected by NAc-DBS (stimulation by stimulus type ANCOVA; DBS: F_1,7_=1.9, p=0.205; **Supplementary Table 8, Supplementary Fig. S3a**), nor were the number of missed encoding responses (stimulation by stimulus type ANCOVA; DBS: F_1,7_=2.3, p=0.170; **Supplementary Table 9, Supplementary Fig. S3b**). History of stimulation (*i.e.*, whether a given stimulus had been presented during an OFF or ON period; OFF_history_ or ON_history_) did, however, significantly alter RTs for subsequent R responses during recognition (stimulation by stimulus type ANCOVA DBS: F_1,7_=12.5, p=0.009; **Supplementary Table 10, Supplementary Fig. S3c-d**), but not for subsequent K or missed responses. R responses that pertained to pictures presented during DBS were faster than during OFF periods.

### Stimulation history does not modulate subsequent stimulus emotional ratings

In light of the role of NAc in appetitive, positive affective states^34^, it is possible that emotionally neutral stimuli presented during NAc-DBS undergo subjective modification of their affective value, which could in turn lead to their better retention in memory. To test for this, 3 patients completed an emotional rating task on all presented images following recognition testing. Patients rated each image in terms of arousal (on a scale from 1 to 9, non-arousing to most arousing) and valence (from 1, most negative valence, to 9, most positive). Ratings for images presented during encoding were separated according to whether they were presented during OFF periods (1, 2, 4 and 6) or during NAc-DBS (periods 3 and 5; ON). For each patient, ratings of arousal and valence for each stimulus type (neutral, pleasant, oddball) separately showed no modulation by stimulation history (all Mann-Whitney U tests *P* > 0.13; **Supplementary Table 11**). Thus, history of stimulation did not modulate the subsequent affective appraisal of stimuli, making this an unlikely explanation for improved memory performance.

### NAc-DBS enhances spatial memory accuracy

**Figure 3.**
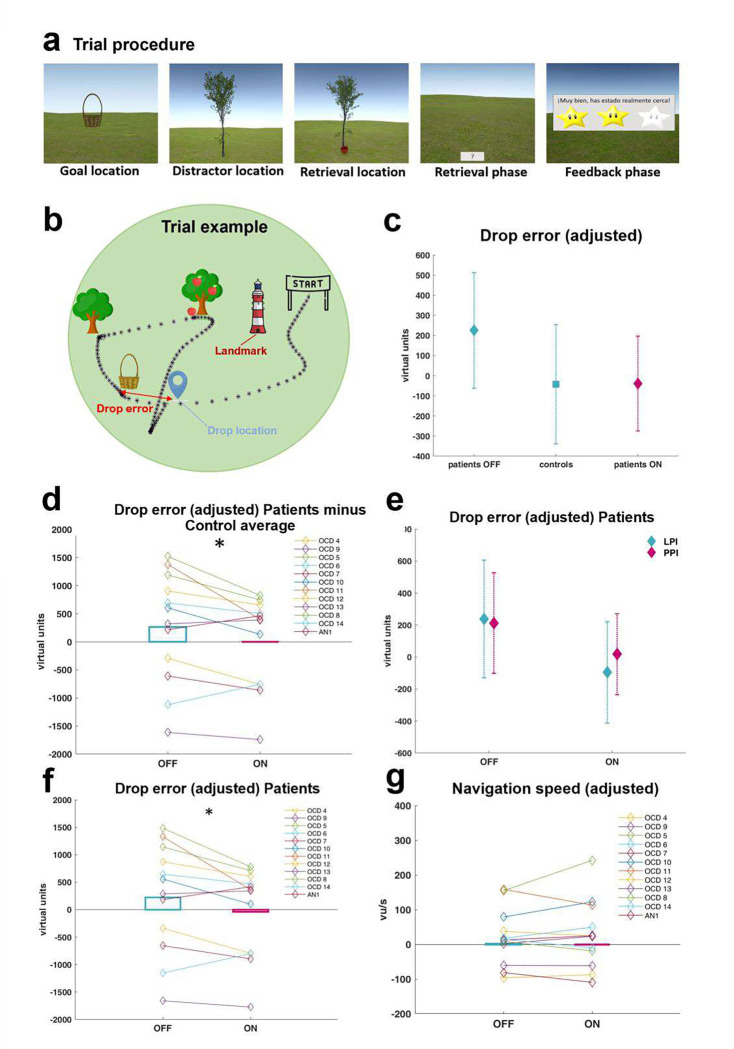
NAc-DBS enhances spatial navigation performance for both pure path integration and landmark-supported path integration (Exp 2). **a.** Each path-integration trial consisted of navigation toward the goal location (basket), toward a distractor location (tree without apple) and finally toward the retrieval location (tree with apple). Afterwards, subjects were asked to return to the remembered goal location within 60 seconds (retrieval phase) and “dropping” the apple at the remembered goal location via a button press. Visual feedback informed the subjects about their response accuracy (feedback phase). **b.** The navigation path of one patient’s trial is plotted as black dashed line. The drop error is calculated as the Euclidean distance between the drop location and the correct goal location. Half of the trials included a lighthouse serving as landmark. **c-d.** Average drop errors, adjusted for a linear learning effect across subtasks, for healthy control subjects and patients OFF *vs*. ON DBS. The performance difference between patients and healthy control subjects was significantly reduced by NAc-DBS. **e-f.** In both subtasks (*i.e.*, landmark-supported path integration and pure path integration) patients’ drop errors were significantly improved in ON *vs*. OFF periods. **f.** Adjusted drop errors for patients OFF *vs*. ON DBS, averaged across navigation subtype. **g.** NAc-DBS did not influence the speed of navigation, measured as virtual units/second; Error bars represent standard errors of the mean; * p<0.05

In Exp 2, the modulation of spatial memory by NAc-DBS was tested with a virtual path integration task (Fig. 3a–b). In contrast to Exp 1, patients that participated in Exp 2 were under chronic DBS treatment (for up to 9 years), with testing preceded by a 2h “wash-out” period of no stimulation. Comparing spatial memory performance between DBS patients and healthy control subjects at baseline (periods 1 and 2 combined) showed significantly higher drop errors in OCD patients than control subjects (t_22_=2.4; p=0.026). Limiting analyses to periods 3 to 6 in the patient group, drop error was significantly lower during ON vs. OFF periods (t_11_=2.46, p=0.032), confirming that NAc-DBS improved path integration performance. For subsequent analyses, all dependent variables were corrected for a learning effect across blocks, by estimating over all patients and subtracting a linear fit before averages were calculated across OFF (4&6/3&5) and ON (3&5/4&6) periods. We calculated the difference between each patient’s average performance per period and the overall performance of the control group in the corresponding period. In other words, for each of the experimental periods, we calculated the average drop error across all healthy controls to examine the extent to which each DBS patient deviated from this healthy control mean. We found that this deviation was significantly reduced in periods when DBS was ON vs. OFF (t_11_=2.4; p=0.034; Fig. 3c–d; **Supplementary Tables 12–13**; **Supplementary Fig. S4**). That is, when DBS was switched on, patients’ performance approached those of healthy control subjects.

Next, we analyzed the effect of NAc-DBS on spatial memory separately within the patient group, including the two different subtasks. A subtask (PPI, LPI) by stimulation (ON, OFF) ANCOVA showed a significant main effect of stimulation (F_1,10_=5.4; p=0.042; η_p_^2^=0.35; Fig. 3e–f), with a stimulation-related reduction of drop error, evident in 9 out of 12 patients. The absence of a significant interaction indicated that this spatial memory improvement was independent of the type of navigation subtask.

### NAc-DBS does not affect speed of navigation

Performance in this task also depended on the patients’ ability to use the joystick and skilfully navigate through the virtual environment, especially with the limitation of 60 seconds during the retrieval phase. We calculated speed of navigation in terms of virtual units (vu) per second to test whether NAc-DBS influenced the patients’ motor skills, but no significant difference was found between navigation speed during ON vs. OFF periods (F_1,10_=0.1; p=0.746; Fig. 3g). Furthermore, NAc-DBS did not influence the absolute response time during the retrieval phase, *i.e.*, the time between collection and “drop” of the apple (F_1,10_=0.9; p=0.342; **Supplementary Fig. S5a**). We also examined excess path length, a measure of efficiency in task performance, and its modulation by NAc-DBS. In contrast to drop error, which is a measure of path integration, excess path length indexes executive functioning and action planning. Efficient task performance^35^ involves making less tortuous routes to the goal, which can be calculated as the total distance covered on the way from apple to drop location minus the correct path length, i.e. the shortest distance from apple to basket location. Excess path length was not different between ON and OFF periods (F_1,10_=1.17; p=0.305; **Supplementary Fig. S5b**) suggesting that executive and action planning components of task performance were unaffected by NAc-DBS. Therefore, the observed DBS-dependent improvement of drop error scores appears to reflect an improvement of spatial memory, rather than a general phasic enhancement of cognitive or motor abilities.

### Memory improvement is unlikely to reflect transient clinical benefit

Impairments in several cognitive domains, including attention, learning and memory, and executive functions have been observed in OCD^36^ and AN^37^. However, it is unlikely that long-term memory improvement is simply attributable to a NAc-DBS-evoked improvement in OCD symptoms. For both Exp1 and 2 cohorts, longitudinal assessments of the psychiatric effects of stimulating different electrode contacts in the striatum, including NAc, were available for six patients, respectively^38^. Long-term stimulation of three months was applied with an amplitude of 4.5V (as opposed to 3.5V in the visual memory task) and monopolar at the most ventral NAc contact C0) as opposed to bipolar between the two NAc contacts C0 and C1). In these six patients, percent improvement in the Yale-Brown Obsessive-Compulsive Scale (YBOCS) after long-term stimulation did not correlate with improvement of memory for oddball pictures induced by transient NAc-DBS (Kendall’s tau=−0.2; p=0.573), neither with improvement of spatial memory (Kendall’s tau=−0.07; p=0.851). Furthermore, a generalized enhancement of cognitive functioning induced by NAc-DBS is unlikely given the absence of an effect on response times during visual memory encoding (Exp 1) and virtual navigation (Exp 2) and the selective memory benefit for oddball and (marginally) neutral stimuli, but not for positive stimuli in Exp 1.

### Neuroanatomical spatial mapping of DBS-induced memory enhancement

**Figure 4.**
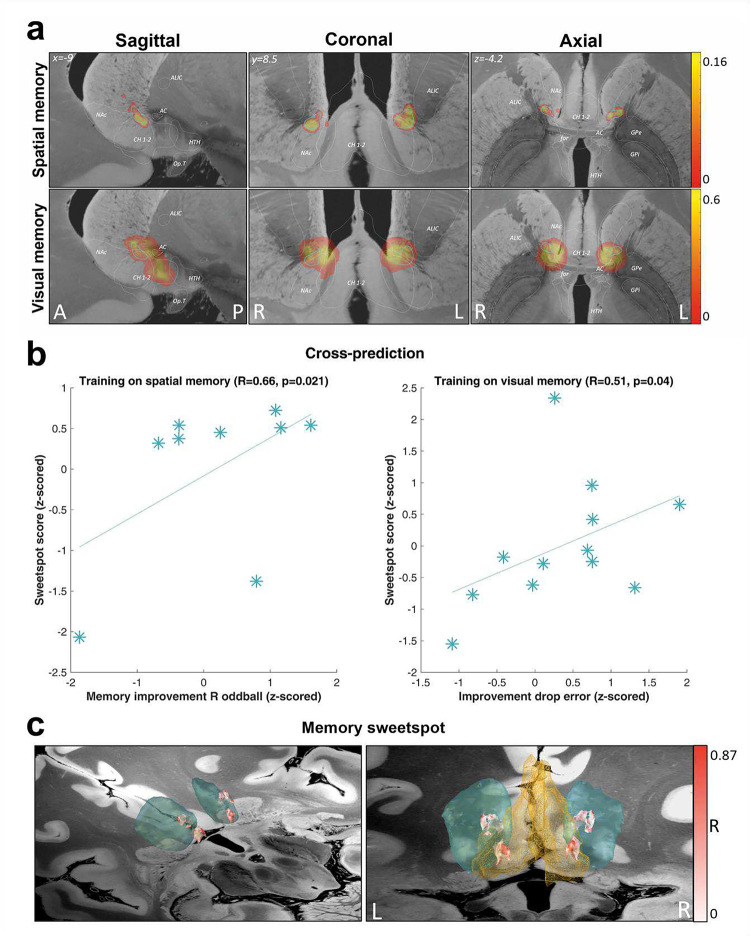
“Sweetspot” for memory improvement. **a.** Voxel-wise correlations (Spearman) between e-fields and memory improvement reveal a region between the postero-dorso-medial NAc and the medial septum, vertical limb of the diagonal band of Broca (Ch1–2) associated with DBS-induced memory outcome, which is highly similar across the spatial (upper panel) and the visual (lower panel) memory task. The overlap between both sweetspots is indicated by the blue outline. Color bars depict R values after spatial smoothing. **b.** Overlaps between stimulation sites and sweetspot from one task correlated significantly with memory improvements in the other task and vice versa. **c.** 3D visualization of the sweetspot (white to red) based on z-scored improvement scores pooled across both tasks. The medial septum and vertical limb of the diagonal band of Broca (CH1–2) are outlined in yellow, and NAc in green. AC: anterior commissure; for: fornix; Gpe: Globus pallidus externus; GPi: Globus pallidus internus; HTH: hypothalamus; Op.T: optic tract

The precise anatomical loci of DBS for optimal management of psychiatric symptoms are currently under study^39, 40^, but the neuroanatomy and neurobiological mechanisms underlying cognitive effects of NAc-DBS remain unexplored. Brain regions in close proximity to the NAc DBS target, such as the fornix or the cholinergic basal forebrain nuclei, are, like the accumbens, known to have modulatory effects on memory and hippocampal activity^17, 41, 42^. We therefore scrutinized differences in anatomical sites of stimulation to delineate “sweetspots” within this area, that is, precise anatomical loci optimal for memory improvement. Volumes of activated tissue (VATs) were medtronic3.5V using a finite element method (FEM)^43^. To map memory improvement to anatomical space, the electric fields (E-fields), thresholded at 0.2 V/mm in each voxel were correlated with the corresponding improvement scores. Specifically, improvement scores were absolute differences of R hit minus false alarm rates for oddball pictures ON minus OFF DBS for each patient in Exp 1, and the difference of drop errors (adjusted for linear trend) OFF minus ON DBS for each patient in Exp 2, both variables z-scored to allow for comparability across both studies. Strikingly, the memory-enhancement effect of DBS localized to the same area for both memory improvement scores, at the border between the postero-dorso-medial part of the NAc and the medial septum/vertical limb of the diagonal band of Broca (areas Ch1–2) (Fig. 4a). The high degree of spatial overlap between both sweetspots (highlighted by the blue outline in Fig. 4a) suggests that the same focal area is linked with memory enhancement *in both* studies, even though different types of memory (encoding of visual pictures *vs*. path integration) had been investigated and at different stages during the course of DBS treatment.

Indeed, it was possible to cross-predict DBS-induced memory benefit in one study based on the sweetspot derived from the other study and *vice versa* (Fig. 4b). Thus, the degree of overlap between stimulation site and sweetspot (associated with enhanced recollection of oddball pictures) correlated significantly with the decrease in drop errors in Exp 2 (Spearman rho=0.51; p=0.040). Likewise, a higher overlap between stimulation sites and the sweetspot trained on spatial memory enhancement correlated significantly with increased hits minus FA scores from Exp 1 (Spearman rho=0.66; p=0.021). Based on z-scored improvement scores, we also calculated a sweetspot across both tasks (*N*=21), which confirmed the location from the individual results (Fig. 4c).

### Memory enhancement following sweetspot stimulation

**Figure 5.**
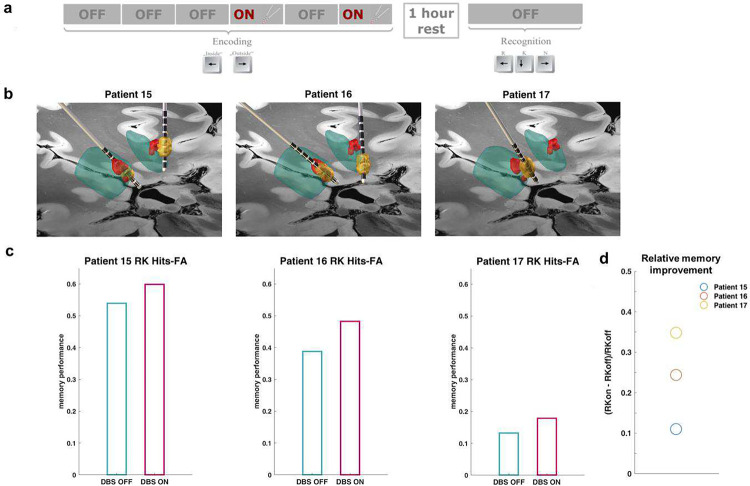
Sweetspot stimulation enhances memory (Exp 3). **a**. As in Exp 1, in each of 6 periods, emotionally neutral and positive stimuli, and infrequent oddball stimuli, were presented. The stimulator was set to ON during the 4^th^ and 6^th^ periods. **b.** Electrode positions for the 3 patients (left to right, Patients 15, 16 and 17) with estimated VAT from bipolar stimulation at 130 Hz, 3.5 V, 60 μs shown in yellow, memory sweetspot in red, and NAc in green. A 7 tesla ex vivo 100-micron T1 scan serves as background template (https://openneuro.org/datasets/ds002179/versions/1.1.0;). **c.** Relative to DBS OFF periods (1,2,3&5), encoding during DBS ON periods (4&6) enhanced the probability that stimuli would later be correctly recognized, pooled over all stimulus types and subsequent recollection (R) and familiarity (K) responses, in all 3 patients. **d**. Relative memory improvement for the 3 patients, calculated as (memory performance ON – memory performance OFF)/memory performance OFF DBS.

A further 3 patients undergoing NAc-DBS for OCD (Table 1) performed the memory task described in Exp 1, but in this experiment, the stimulated electrode contacts were selected such that the bipolar VAT was nearest to the sweetspot derived from Exp 1 and 2. Physical overlap between the closest VAT and the sweetspot was evident in the left electrode of all 3 patients. Note that Patient 17 underwent relocation of the left electrode 4 days prior to Exp 3; the right electrode had been chronically stimulated for 6 years and was therefore not stimulated. Furthermore, given that stimulation order had been fixed in Exp 1 (ON periods were 3^rd^ and 5^th^ for all patients), in this experiment stimulation was delivered during the 4^th^ and 6^th^ periods. Replicating the observations from Exp 1, but now with targeted sweetspot stimulation and a different stimulation order, all 3 patients show memory enhancement (Fig. 5), with a median relative memory enhancement of 24.4% comparing subsequently correct R and K responses during stimulation *vs*. off stimulation.

### Structural connectivity places the stimulation site within a hippocampal-VTA circuit

**Figure 6.**
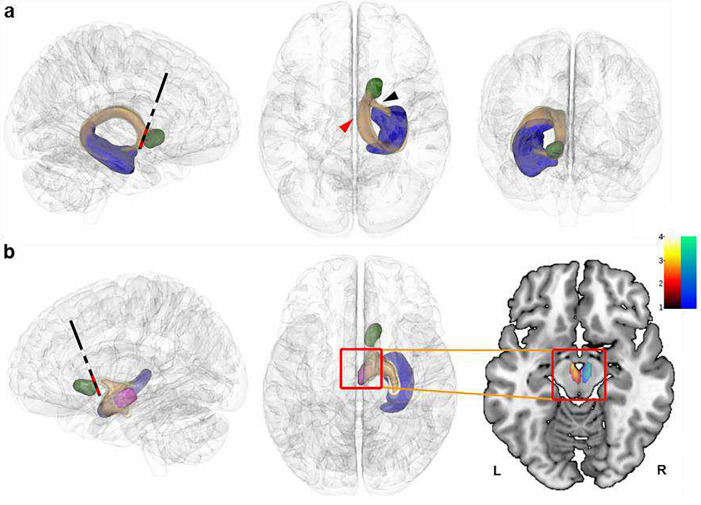
Anatomical connectivity between nucleus accumbens stimulation site and hippocampus. **a.** Segmented right NAc (green) and hippocampus (blue) are shown on a transparent brain seen from sagittal (left), axial (middle) and coronal (right) perspectives. In sagittal view, the position of the right stimulating electrode is indicated as per Fig. 2. The two major fibre bundles connecting NAc and hippocampus, the fornix dorsally plus a ventral pathway, are indicated in beige and highlighted in axial view by red and black arrows, respectively. **b**. The VTA mask is shown (purple). Beige surfaces demonstrate results of probabilistic tractography using stimulation site as seed, and hippocampus as a target. On the right, an axial MRI slice in MNI space is shown, indicating the number of patients that show agreement in the probabilistic tractography map within the VTA. Color bars represent the overlap between individual patient reconstructions (number of subjects) in left (L) and right (R) hemispheres.

Remember, but not Know, retrieval judgments are considered critically dependent on hippocampus (^21, 44^ but see ^23^), indicating the likely engagement of hippocampus in the enhanced recollection, but not familiarity-based, recognition we observe. To determine a neuroanatomical substrate for NAc-hippocampal co-operation, we measured structural connectivity between the NAc stimulated site and hippocampus by applying probabilistic tractography to pre-operative diffusion weighted brain images from 7 OCD patients. The stimulated NAc site connected to the hippocampus not only via the fornix, but also via a ventral pathway (Fig. 6a). In addition to these two pathways, the NAc is also thought to be central to a circuit which enhances levels of dopamine in the hippocampus via engagement of the ventral tegmental area (VTA)^2^. We therefore next tested for an overlap between projections between NAc and VTA, and hippocampus and VTA, and successfully identified overlap in the VTA in 6 of the 7 patients (Fig. 6b).

### Local and distant effects of NAc-DBS on neuronal activity

**Figure 7.**
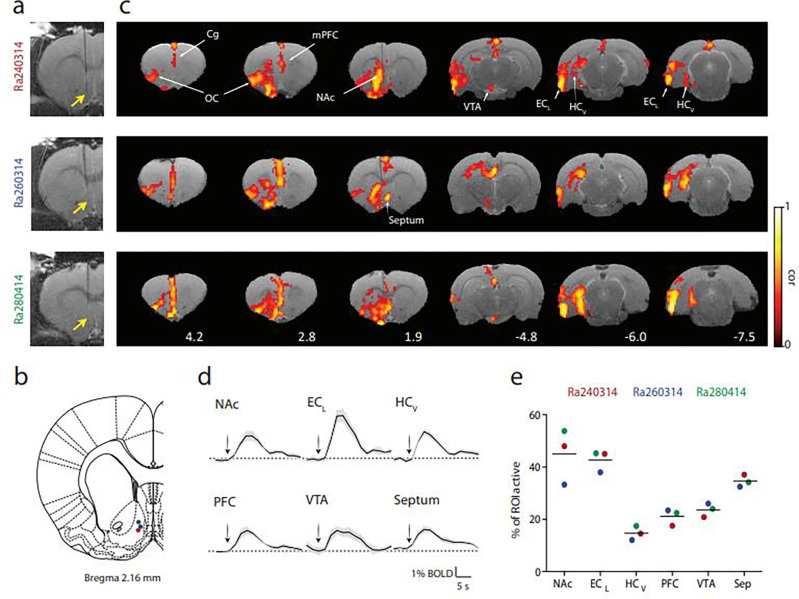
Activation of brain regions induced by DBS of the shell of the NAc in rats. **a**. High resolution anatomical images (T2-weighted) showing the location of the DBS electrode in three different animals. The electrode is visualized as a thin vertical line crossing the corpus callosum, with the yellow arrow pointing to its most ventral location. **b**. Location of the implanted electrodes mapped on the Paxinos and Watson rat brain atlas. Colors denote the identity of the individual animals as in a. **c**. Thresholded functional maps (p < 0.001, cluster size 14) of the three animals (in rows) overlaid on anatomical T2-weighted images, showing brain activation in DBS ON periods. Color-code denotes the correlation coefficient of the BOLD signal with the stimulation protocol. Numbers on the images of the lower row indicate distance from bregma in mm. **d**. BOLD signal time courses evoked by DBS in different regions. **e**. Volume of activated regions as percentage relative to the total volume of the structure. Cg: cingulate, EC_L_: lateral entorhinal cortex, HCv: ventral hippocampus, mPFC: medial prefrontal cortex, NAc: Nucleus accumbens, OC: orbitofrontal cortex, PFC: prefrontal cortex, Sep: septum, VTA: ventral tegmental area. Note that signal drop-out precludes effective imaging of the medial EC.

Structural connectivity does not, however, inform about the functional correlates of stimulation. Functional MRI acquisition during active DBS is limited in human patients. In Exp 4, we therefore employed an animal model to measure whole-brain functional correlates of DBS in the equivalent anatomical site in rats. In the context of fMRI scanning, anesthetized rats underwent left unilateral, bipolar stimulation of the NAc shell (*i.e*., the same structure targeted in our patients) using parameters equivalent to those used clinically: 130 Hz stimulation at 150 μA (~ 3.5 V) and pulse width 60 μs (Fig. 7a–b). Responses in 3 animals were measured and we inspected the similarity of evoked activity between animals. Stimulation for 4 s evoked a rise in blood oxygen level-dependent (BOLD) signal in several structures critical for memory function, including the NAc itself, ventral hippocampus and VTA, confirming functional relevance of our structural connectivity findings (Fig. 7c–d). Medial and orbitofrontal cortices were also activated, as well as the medial septum. The magnitude of response was highly consistent across subjects (Fig. 7e).

## DISCUSSION

Memory function is modulated by several structures that project to the hippocampus^45^, including the mesolimbic dopamine system^8, 9, 11, 33, 46^. We hypothesized that stimulating the nucleus accumbens in patients undergoing DBS would influence memory. We confirmed this hypothesis by showing NAc-DBS evoked memory enhancement in humans. In contrast to the few preexisting human NAc-DBS studies assessing long-term changes in cognitive abilities^14, 15^, we explicitly modulated neural activity in a placebo-controlled block-design to examine the effects of transient NAc-DBS both in the immediate postoperative phase and after chronic therapeutic DBS. This also has the advantage of avoiding practice effects and poor test-retest reliability when the same neuropsychological test is repeatedly assessed over time. Two previous studies have shown memory benefits after long-term NAc-DBS and in both studies, memory scores were not significantly related to psychiatric improvements, in agreement with present results. The first study in 10 patients with major depressive disorder (MDD)^14^ reported significant improvements in verbal and visual spatial memory after 1 year of bilateral NAc-DBS (at the same stimulation target used in the current study). In the second study, a group of 10 OCD and 11 MDD patients who received DBS to the anterior limb of the internal capsule/ventral striatum, a site adjacent to the current target, showed significant improvements in verbal recall during chronic stimulation^15^. By contrast, longitudinal studies of memory in treatment-resistant depression patients undergoing DBS of the ventral anterior limb of the internal capsule (vALIC) show either no effect on verbal or visuospatial memory^47^ or a decline in episodic memory scores of the Autobiographical Memory Inventory Short Form induced by DBS treatment (although this decline was observed relative to healthy controls and not treatment-resistant depression patients not undergoing DBS)^48^. Patients in our second study had already been stimulated for a chronic period of up to 9 years, and although chronic stimulated contacts and stimulation settings differed from those applied in our study, half of the patients had been chronically stimulated at NAc contacts and most of them with higher voltages/currents. This is remarkable, as we observed a transient memory effect of Nac-DBS even in these patients, suggesting that little to no habituation to DBS effects on memory occurred over time.

Our first experiment revealed significantly enhanced encoding of salient visual stimuli following transient NAc-DBS in the acute postoperative phase and a trend for improved encoding of neutral pictures. Encoding effects were reflected in higher hits minus false alarm rates of conscious recollection, but not of familiarity judgments, consistent with an upregulation of hippocampal processing^21, 44^. The enhancement of memory for salient events by NAc-DBS is in line with a model whereby the hippocampus and dopaminergic mesolimbic system gate entry of novel stimuli into long-term memory^2^. Conceivably, DBS of the NAc facilitates the encoding of salient stimuli by further reinforcing a dopaminergic loop, which is already activated by the perception of an unexpected stimulus. It should be noted that memory enhancement for neutral pictures in Exp 1 also reached trend level significance (p*=*0.06), which may simply be a limitation of sample size, raising a possibility that NAc-DBS renders these stimuli as more salient in the memory encoding process, putatively via increased release of dopamine within the hippocampus and neocortex.

Numerous studies implicate a striatal contribution to other types of memory, including spatial memory^12, 49^. Our second experiment replicated and extended the finding of improved memory ability from Exp 1, this time testing patients’ spatial memory accuracy with and without stimulation, after a chronic period of DBS therapy. Memory enhancement in this study was independent of the subtype of path integration, *i.e.*, in the presence or absence of a landmark. In both Exp 1 and 2, the effects of DBS are unlikely to be due to a general enhancement of cognitive or executive functioning, nor did these effects correlate with longitudinal clinical improvement following long-term accumbens stimulation. In an additional analysis, we delineated an anatomical sweetspot at the border of the postero-dorso-medial NAc and the cholinergic medial septal nuclei (Ch1–2) associated with memory benefit in both studies. Stimulation overlaps with the sweetspot derived from one study could cross-predict DBS memory enhancement in the other study.

Convergent findings from our patient tractography and rodent fMRI analyses support a central role for NAc in a proposed hippocampal-VTA circuit, that further involves medial and orbitofrontal cortices, important for enhancing memory for salient events^2^, and overall suggest a role for NAc-DBS in strengthening the functional coupling in this memory network. Consistent with this interpretation, we have previously shown that potentiating the input from rat EC to hippocampus in the context of fMRI scanning results in the formation of a strongly coupled functional network including hippocampus, medial-prefrontal and orbitofrontal cortices and NAc^50, 51^. Critically, under NAc inactivation, the coupled network disintegrates, highlighting the key role of NAc in maintaining a hippocampal-mesolimbic-PFC circuit^52^.

The mechanisms of action of DBS remain incompletely understood^53, 54^. However, it is clear that stimulation leads to both local and remote effects^53^, both of which can be observed in our rat fMRI data. Locally, the increased BOLD response we observed in NAc was consistent with a previous study in OCD patients showing that NAc-DBS immediately before fMRI scanning normalized (increased) NAc activation during reward anticipation^5^, although we note the potential of local artefacts in functional T2* images from DBS. With respect to remote effects, NAc-DBS-evoked activation in medial and orbitofrontal cortices was in keeping with our previous demonstration that cortical projections from the stimulation site were strongest to ventromedial prefrontal and orbitofrontal cortical areas^4^. Our patient tractography analyses showed that the stimulation site was anatomically connected to the hippocampus, with which it shares confluent projections to the VTA. FMRI results from the rodent experiment added functional validity to these anatomical findings by showing that stimulating this same NAc region with the same stimulation parameters activated both hippocampus and VTA. This hippocampal response was ventrally located, in agreement with the anatomical and functional relationship between NAc shell and ventral hippocampus^11^. Thus, despite potential between-species differences in our stimulation protocol, such as much clearer anatomical demarcation of the NAc shell in rodents compared to humans, potential differences in volume of tissue activated by rodent *vs.* human stimulation protocols, and likelihood of stimulation of the same surrounding brain structures due to between-species anatomical differences, results from all of our three experiments converged on the same brain circuit that could mediate NAc-DBS induced memory enhancement.

The electrode placement in the postero-ventro-medial NAc (*i.e.,* shell) was in close proximity to the more medially located basal forebrain cholinergic nuclei (medial septum and vertical limb of the diagonal band of Broca) which provide most of the cholinergic input to the hippocampus^16^. In rats, DBS of the cholinergic medial septal nucleus restores spatial memory after partial pharmacological lesions of medial septal cholinergic neurons^55^, which is also associated with increased hippocampal theta activity^55^. We have shown that rodent NAc stimulation engaged the medial septum and our sweetspot analysis equally encompasses this region, indicating that increased cholinergic input to the hippocampus may also be driving the memory improvement described here. Thus, stimulation of this memory sweetspot could potentially influence hippocampal activity by engaging two neuromodulators – dopamine and acetylcholine.

Previous studies demonstrating memory enhancing effects of DBS have stimulated primary hippocampal input/output pathways: the fornix and entorhinal cortex (EC). Fornix-DBS has been reported to induce autobiographical memory flashbacks and improve recollection in a patient undergoing lateral hypothalamic DBS for obesity^56^ and in 4 cases of epilepsy (without statistical evaluation)^57^ for visual, but not verbal, memory. Despite initial promise of a first clinical trial for the treatment of Alzheimer’s disease (AD) by fornical DBS^41^, subsequent phase II trials have not shown benefit, on a group level^58^. EC-DBS was shown to improve spatial memory in 7 patients with epilepsy^59^, although in further epilepsy patients performing similar tasks, the same stimulation parameters provoked memory impairment^54, 60, 61^. More recent work, however, showed that microstimulation in the right entorhinal area during learning in a person recognition task significantly improved subsequent memory specificity^62^, and that, more generally, stimulation of right entorhinal white matter, but not left-sided or gray matter stimulation, improves visual memory performance^63^. Other approaches to improving memory in epilepsy patients have involved time-locking DBS to immediately after visual stimulus presentation (in the case of amygdala stimulation^64^) or using a read-out from on-going electrophysiological recordings to trigger stimulation (of lateral temporal cortex^65^). The approach for enhancing memory described here, however, does not require external or feedback triggering.

In contrast to fornix and EC targets, here we provide evidence for an alternative approach associated with memory enhancement: targeting a system that may activate the hippocampus through modulatory channels. Our observations are relevant to recent attempts to employ DBS as a technique to treat memory loss in dementia for two reasons. First, evidence is emerging that NAc-DBS improves memory after long-term stimulation (yet to be shown for EC or fornix stimulation) in psychiatric patients, with this memory-enhancing process appearing to be independent of its antidepressant or anti-obsessive-compulsive effects. Second, the system we target, which modulates the hippocampus via parallel channels, particularly the putatively dopaminergic one, may be less subject to neurodegeneration in AD, the leading cause of dementia, than the fornix and EC, themselves. As mentioned above, NAc-DBS may normalize functional responses in this structure^5^. Such restoration of local function could be essential to therapeutic benefit and would be difficult to achieve if the stimulated area is severely degenerated. This caveat equally applies to a trial of DBS of nucleus basalis of Meynert (NBM). The NBM has long been known to be a site of extensive degeneration in AD^66^ even at early stages. NBM-DBS in 6 patients with mild to moderate AD^67^ produced slightly less worsening of clinical status than would be expected after one year^67^. Even if EC-DBS in epilepsy patients had shown consistent memory enhancing effects, the atrophy of EC observed in the earliest stages of AD (which primarily involves cell-layers projecting to hippocampus^68^) may render this site, like fornix and NBM, a suboptimal target for AD. By contrast, the NAc may be relatively preserved in AD^69^, although we note that any cholinergic contribution from stimulating the medial septal area encompassed by the memory sweetspot described here is likely to be diminished or absent in patients with AD.

By applying focal electrical stimulation to the human NAc, we provide direct evidence for an on-line role for this structure in episodic memory enhancement, which in humans has until now only been indirectly inferred from correlative neuroimaging data. The enhancement in subsequent recollection of visual stimuli and spatial navigation performance across both landmark-supported and pure path integration tasks induced by NAc-DBS implies engagement of a hippocampal-dependent process. This is supported by patient diffusion-weighted imaging data and rodent fMRI responses demonstrating structural and functional connectivity between the stimulated site and hippocampus. Moreover, these analyses indicate membership of the stimulated NAc site to a circuit comprising accumbens, hippocampus, medial and orbitofrontal cortices and VTA, providing direct support, in humans, for a model of this circuitry in upregulating episodic memory^2^ for salient events and spatial memory. Our observations provide mechanistic insights and the strong inferential power of focal, transient neuromodulation, to support observations of memory enhancement following long-term NAc-DBS that appears to be de-coupled from psychiatric improvements. These observations provide an empirical and mechanistic basis for considering NAc-DBS a potential therapeutic avenue for patients with memory impairment.

## ONLINE METHODS

### Human studies.

#### Participants.

Participants in Exp 1 comprised 8 patients suffering from treatment refractory OCD (20–50 y; average: 34.8 y; 3 female), and one patient with treatment-resistant anorexia nervosa (female; age 37; Table 1). This patient had a preoperative Body Mass Index (BMI) of 15.4 and the following scores on psychiatric scales: Bulimic Investigatory Test, Edinburgh, Severity subscale and symptoms subscale (BITE) Symptoms 26, Severity 16; and the Bulimia Test-Revised (BULIT-R) score of 110.

The OCD patient control group (not undergoing DBS) comprised 9 patients (21–50 years; average: 34; 2 female; **Supplementary Table 2**). One control patient (cOCD3) has a left-sided pupil lesion. All other participants were free of visual impairments or color blindness.

Fifteen patients suffering from treatment refractory OCD and one patient with treatment-resistant anorexia nervosa (patient AN1; age 36; female), who had all been implanted for NAc-DBS therapy, completed Exp 2. The OCD patients were under the care of three different hospitals in Spain. Two OCD patients had to be excluded from Exp 2 due to poor performance in the task, i.e. due to exceptionally high drop errors or response times at baseline, deviating by more than two standard deviations from the average. Another OCD patient had to be excluded for pronounced cognitive side effects from medication (unable to perform the task), so that 12 patients (aged 20–54 years; average: 34.5 years; 3 female) were included in the analyses. Six patients completed both Exp 1 and 2 (Table 1). Twelve healthy control subjects matched in gender and age, recruited within two research centres in Madrid, participated in Exp 2. A further 3 patients with OCD undergoing NAc-DBS participated in Exp 3.

All patients and control participants provided written informed consent. Both studies had full ethical approval from the Hospital Clinico San Carlos, Hospital Universitario La Princesa and Universidad Politécnica de Madrid ethics committee. OCD patients 1–3 and 5 took part in the 2-year longitudinal study, which also had approval from the Hospital Clinico San Carlos Ethics Committee and was registered at clinicaltrials.gov under trial name “Deep brain Stimulation in Obsessive-compulsive Disorder: Randomized, Double-blinded Clinical Trial (10/131)”, registration number NCT03217123. Approval for intervention with DBS in patient AN1 was proportioned on compassionate grounds from the Spanish Medication Agency (AEMPS), registration number 544/16/AE.

#### Neurosurgical procedure

Full operative details for HCSC patients have been recently described^4^. In brief, a Medtronic Stealth Station Treon navigation system (Medtronic Minneapolis, USA) was used to place the nucleus accumbens target at coordinates reported previously^3^. For patients OCD1–6 and AN1, a trajectory was planned to reach the target point at the nucleus accumbens close to the bed nucleus of the stria terminalis (distal electrode contact), and for placing the rest of the contacts of a Medtronic Model 3391 stimulating macroelectrode (four 3.0-mm contacts in total, 4.0-mm spacing between contacts; 1.5-mm spacing after most distal contact) at several points along the striatum avoiding the ventricles. For patient OCD7, the same target co-ordinates were used, but the insertion trajectory followed the internal capsule, just lateral to the caudate. The electrodes inserted in this patient and in patients OCD8 and 13 were Boston Scientific (Marlborough, MA, USA) DB-2201 (unsegmented octopolar model; 8 1.5-mm contacts in total, 0.5-mm spacing between contacts; 1.2-mm spacing after most distal contact). For patients OCD8–17, electrodes were implanted so as to target the internal capsule, ventral striatal and NAc with consecutive contacts^70^. Patient AN1 also underwent bilateral electrode implantation to ventral midbrain, but these electrodes were inactive prior to, and during, the testing session. For all other patients, preoperative mounting of the stereotactic frame (Leksell Coordinate Frame G; Elekta Instrument AB, Stockholm, Sweden) under general anesthesia was followed by a computed tomography (CT) scan. The anterior and posterior commissures were identified using axial three-dimensional T1-weighted inversion recovery axial MRI. Images were transferred to a neuronavigation station (Brainlab AG, Munich, Germany) and used to place the nucleus accumbens target as described above.

After microelectrode recording, the macroelectrode was implanted at the determined target. Final electrode position was verified by post-operative computed tomography (CT) and 1.5T MRI.

#### Deep-Brain Stimulation Protocol

Exp 1 was conducted up to six weeks post-implantation, with stimulator OFF during this period. Exp 2 took place after a chronic period of therapeutic stimulation, ranging from one month to 9 years. Stimulators were turned off two hours prior to testing and only turned on during two blocks of the spatial memory task. For patients OCD1–6, 9–12 and 14–15, bilateral NAc-DBS was delivered via a constant current stimulator as square pulses, using a Medtronic N’Vision Model 8840 Clinician Programmer and Software application card model 8870 (programming platform for Medtronic neurological implantable therapy devices) for programming the parameters of the Activa PC neurostimulator model 37601. Bipolar stimulation at 3.5 V between the 2 most distal contacts (negative polarity most distal) was delivered at 130 Hz (pulse width 60 μs). DBS was only applied during periods 3 and 5, with the onset of stimulation 10 s before run start, with an increase from 0–3.5 V ramped over 4 s at onset. For patients OCD7, 8 and 13 and AN1, DBS was programmed with a Boston Scientific Clinical Programmer. As this is current-clamped stimulation, electrode contact impedance was measured, and current delivered to achieve a voltage of 3.5V (voltage increase from 0–3.5 V between periods was again ramped over 4 s at onset). Exp 3 was conducted in the week post-implantation, with DBS programmed with a Boston Scientific Clinical Programmer as described above. For patients OCD15 and 16, bipolar stimulation at 3.5 V between the 2 contacts nearest to the memory sweetspot (negative polarity most distal) was delivered at 130 Hz (pulse width 60 μs). Patient OCD17 had undergone chronic stimulation for the preceding 6 years, with replacement of the left electrode to a more medial location 3 days prior to performing Exp 3. To eschew potential interpretability issues arising from simultaneously stimulating acutely placed and chronic electrodes, stimulation during Exp 3 in this patient was left unilateral.

#### Exp 1. Experimental Protocol:

The study consisted of an incidental encoding session followed one hour later by a surprise recognition memory task.

##### Stimuli.

During encoding and recognition, 3 types of pictures were presented: emotionally neutral, emotionally positive and visual perceptual oddballs. A total of 156 emotionally neutral pictures were taken from the International affective picture system (IAPS) database^71^, with mean (std) scores of valence = 4.933 (0.602) and arousal = 3.373 (0.625), using a scale from 1–9 and 9 being the most positive valence and most arousing. A further 60 neutral pictures were taken from the Geneva affective picture database (GAPED)^72^ valence = 56.869 (6.350) and arousal = 24.544 (7.558), with ratings from 0 to 100 points; 0 = very negative pictures to 100 = very positive pictures; 0 for no arousal, 100 for highly arousing. Emotionally positive pictures (total of 96) were taken from the IAPS database (scores of valence = 7.201 (0.416) and arousal = 5.731 (0.549)). Perceptual oddball stimuli were photographs of black and white objects (taken from the Hemera Photo-Objects database), by contrast to all other stimuli which were in color (total of 48 oddballs). For each stimulus type, half of the stimuli were randomly selected for each patient to be presented at encoding, with the other half presented as lures at recognition (to avoid any confounding effect of pooling across picture databases, for neutral stimuli half of the IAPS and half of the GAPED pictures were randomly selected separately).

##### Encoding.

Prior to encoding, patients were informed that the task would last approximately 13 minutes and that during this time their stimulator would be turned on for two 2 minute periods, but that they would be blind to stimulator status throughout. During the encoding session, patients were presented pictures on a 15” laptop computer screen and indicated, via button-press, whether the picture pertained to an indoor or outdoor scene (stimulus duration 1500 ms, inter-stimulus interval 2500 ms). Patients viewed 180 pictures, divided in 6 periods, with NAc-DBS applied during the 3^rd^ and 5^th^ periods only. In each 10 second delay between successive periods, one experimenter (B.A.S.) manipulated the Clinician Programmer either actually turning ON/OFF the NAc-DBS or, after the first period, giving the impression of doing so. This experimenter then left the testing room. For patients from HCSC, a further experimenter (J.M.A-C.) was present throughout testing, and was also blind to stimulator status. For each period, 18 neutral pictures (13 IAPS and 5 GAPD), 8 positive pictures and 4 perceptual oddballs were presented (*i.e.,* oddballs had a 13.3% probability of occurrence). Pictures were pseudorandomly presented with two constraints: 1) the first 5 stimuli in each epoch were always neutral, to set the context for perceptual oddball stimuli, and 2) that there was at least one non-oddball stimulus between successive oddballs. Note that our rationale for not counterbalancing which periods were stimulated in Exp 1 was to facilitate analysis of any retrograde or carry-over effect of NAc-DBS into OFF periods that followed ON periods (this would not have been possible had stimulation occurred in the first blocks or the last block). In Exp 3, described below, a further group of patients performed the same task, with stimulation applied in blocks 4 and 6.

##### Recognition.

One hour later, patients performed a surprise recognition memory test. All stimuli shown at encoding were presented, randomly intermixed with an equal number of new lure items (stimulus duration 1500 ms, inter-stimulus interval 2500 ms). Patients were required to make a push-button response to indicate whether they had seen the picture before. Specifically, patients were required to make a “remember” “know”, or “new” decision^20^, with remember responses indicating that the patient could recall elements of the study episode, whereas know responses indicated the patient had a sense of familiarity with the picture without being able to recall the original study episode. NAc-DBS was not applied during recognition. At the end of testing, each patient was asked whether they could recount at which time points during encoding the stimulator had been set to on. No patient was able to correctly identify the two periods of NAc-DBS. Furthermore, no patient reported symptoms of elation on starting stimulation, which we have observed previously in one patient^4^.

##### Emotional rating.

Following recognition testing, the same visual stimuli were presented again to 3 patients (OCD5, OCD7 and AN1) who were instructed to judge the valence and arousal of each image according to 2 self-assessment manikin (SAM) images^73^. Pictures were presented for 1500 ms, followed by a fixation cross (500 ms) and then the valence SAM was presented, requiring a self-paced button-press to indicate 1, most negative valence, to 9, most positive. Once the patient had made a response, a fixation cross again appeared (250 ms) followed by the arousal SAM. A self-paced button-press (a scale from 1 to 9, non-arousing to most arousing) prompted a fixation cross (500 ms), followed by the subsequent picture.

##### Statistics.

For each patient, encoding success – the percentage of subsequently correct remember (R) or familiar (K) judgments (*i.e.,* Hit rate) – was calculated for each stimulus category for each period. False alarm rates for R and K responses (*i.e.,* indicating that a novel lure was previously seen during encoding) was calculated for each stimulus category and was subtracted from the corresponding Hit rate. Stimuli for which there was a missed response at encoding or recognition were removed from analyses. The only exception to the latter was for one encoding period, for one patient (OCD7), where no responses were made to a stimulus type and the recognition for these stimuli was zero. Behavioral data were analyzed using IBM SPSS, version 22^®^. Due to the broad age range of patients from 20–50 years, age was included as a covariate in all statistical tests and all reported *P* values ensuing from t-tests are two-tailed.

#### Exp 2. Experimental Protocol:

Spatial memory performance was assessed using a virtual navigation task. We employed an adapted version of the *“Apple Game”* ^24^, which is implemented via Unreal Engine (Epic Games, version 4.11). In this task, participants navigate through a virtual environment featuring a grass landscape. The radius of the arena is 16,971 virtual units. Each trial consists of four successive steps: navigation to the location of a basket (*“goal location”*), which they are instructed to remember, navigation to a distractor tree, navigation to the tree with an apple (*“retrieval location”*), return to the goal location within 60 seconds and “drop” the apple via a button press (*“drop location”*). A number of stars, from zero to three, is then displayed as feedback about the proximity to the goal location, before the next trial starts. All objects (basket and trees) appear successively and disappear at the time of passage. The task comprised two subtasks, so that in half of the trials a lighthouse served as a landmark, whereas in the other half of the trials no supportive spatial cues were available and participants had to rely on PPI. Four practice trials allowed the participants to get familiar with joystick navigation before a total of 48 trials were completed, with 8 trials per block. Across all patients, the average trial duration was 49 seconds, with a total task duration of 39 minutes on average. The first two blocks served as a baseline and were not included in the analysis, as a practice effect (*i.e.,* markedly improved performance) between period one and two was observed. Stimulation was applied either during periods three and five (7 patients) or during periods four and six (5 patients).

The distance between basket location and distractor tree (leg a: 1600 or 3200 virtual meters (vm)), between distractor tree and retrieval location (leg b:1600 or 3200 virtual meters (vm)), as well as the angle between the two legs (60° or 120°) varied between trials and was balanced across the three conditions BASELINE (periods 1&2), ON (periods 3&5/4&6) and OFF (periods 4&6/3&5). Spatial memory accuracy was quantified by the drop error, *i.e.,* the Euclidian distance between the drop location and the correct goal location. To test for stimulation-based effects on motor abilities, navigation speed (virtual units per second) was examined, as well as the response time, *i.e.,* the absolute time of the retrieval period, which, in contrast to the navigation speed, also includes times without joystick movement.

##### Statistics.

All dependent variables were calculated using Matlab and corrected for a learning effect across periods, by estimating over all patients and subtracting a linear fit before averages were calculated across OFF (periods 4&6/3&5) and ON (periods 3&5/4&6) periods. That is, for each patient, drop error was averaged across all trials within the 6 blocks. These values were then averaged over patients, irrespective of DBS ON/OFF order. The linear fit over the 6 blocks was estimated using the polyfit function (of degree 1) in Matlab. The linear fit (containing 1 value per each of the 6 blocks) was then subtracted from each patient’s 6 block values. In three patients, one trial was missing, due to interruptions or technical problems. Statistical analyses were carried out in IBM SPSS Statistics, version 22^®^. As in the previous experiment, age was included as a covariate and all reported *P* values ensuing from *t*-tests are two-tailed.

##### Longitudinal assessment

OCD patients 1–3 and 5 took part in the 2-year longitudinal study. In brief, patients underwent a trial of three months of stimulation of every contact: 0, 1, 2, 3, plus a sham 3 month period of no stimulation. Order of stimulation trials was randomized across patients. At pre-operative baseline and at the end of each trial, standardized clinical evaluation, including YBOCS scoring, was performed by a psychiatrist who was blind to stimulation protocol. OCD patients 4 and 6 were not enrolled in this trial but underwent 3 months continuous monopolar NAc-DBS prior to psychiatric evaluation.

##### Brain Imaging

###### Pre-operative.

For OCD patients operated at HCSC, a 3T Siemens TRIO system was used to acquire MPRAGE T1-weighted anatomical images with 1 mm^3^ resolution (repetition time (TR), 2300 ms; echo time (TE), 2.98 ms; flip angle (FA), 9°) and echo planar diffusion-weighted images (DWI). DWI acquisition was based on parameters used in previous probabilistic tractography studies of the basal ganglia^74^. Each volume consisted of 40 axial slices of 2.3mm thickness with no interslice gaps and an acquisition matrix of 96 × 96 in a field of view (FoV) of 220 × 100 mm, resulting in 2.3 × 2.3 × 2.3 mm^3^ isotropic voxels (TR, 5800 ms; TE, 103 ms; flip angle, 90°; bandwidth, 2004 Hz/pixel). In order to increase the SNR, we acquired two contiguous sequences of 128 diffusion-weighted images. Each dataset consisted of 64 images with diffusion gradients applied along 64 noncollinear encoding directions for two different diffusion sensitization strengths (*b* = 500, 1000 s/mm^2^), and one additional image with no diffusion weighting (*b* = 0 s/mm^2^). For patient AN1, a 3T Siemens MAGNETOM Prisma system was used to acquire sagittal T1-weighted anatomical images with 0.977 × 0.977 × 0.9 mm^3^ resolution (TR, 700 ms; TE, 12 ms; FA, 120°).

All other patients underwent preoperative 1.5T MRI scanning (General Electric; GEHC, Waukesha, USA) Signa HDxt,; 2D coronal FSE with TR 686, TE 10.65, flip angle 90°).

###### Post-operative electrode reconstruction.

Postoperative electrode localizations of all patients were carried out using the software *Lead DBS*^43, 75^ and the default parameters of its pipeline. In short, postoperative images (CT scans in 13 patients, MRI scans in three patients) were co-registered to preoperative MRI scans and normalized into MNI space (ICBM 2009b NLIN Asym^76^) using Advanced Normalization Tools (ANTs; http://stnava.github.io/ANTs/^94^). When necessary, manual refinements were performed after visual inspection. To correct for a nonlinear deformation of the brain during surgery, a brain-shift correction method, introduced by Schönecker^77^ was applied. Electrodes were pre-localized either manually or using the automatic *Precise and Convenient Electrode Reconstruction for Deep Brain Stimulation* (PaCER)^78^ approach, followed by a manual, refining localization. **Fig. S1** illustrates the electrode placements of all participating patients and **Fig. S6** provides intersections between surrounding anatomical structures and the volumes of activated tissue (VAT), i.e. the approximate surrounding tissue modulated by DBS (estimated using the toolbox *Lead Group*^79^). Briefly, a finite element method (FEM) approach was applied^43, 80^. Using the Iso2Mesh toolbox (http://iso2mesh.sourceforge.net/) a tetrahedral volume is generated to construct a volume conductor model with the conductivity values of 0.33 and 0.14 S/mm for grey and white matter, respectively, which are commonly used^81–84^. Based on this model and on the amplitude of the active electrode contacts, the potential distribution generated by DBS is simulated employing the FieldTrip-SimBio pipeline (https://www.mrt.uni-jena.de/simbio/index.php/; http://fieldtriptoolbox.org). A 7 tesla ex vivo 100-micron T1 scan (https://openneuro.org/datasets/ds002179/ versions/1.1.0;^85^) served as a background template.

###### Sweetspot analysis.

To determine the anatomical site of stimulation associated with optimal memory outcome, instead of binary definitions of VATs, the vector magnitudes of electric fields (subsequently abbreviated with E-fields), thresholded at 0.2 V/mm were used and mirrored to the other hemisphere, which increases statistical power and is current standard practice^79, 86–90^. After excluding voxels covered by less than 30% of all E-fields in the group, patients’ improvement scores were correlated with E-fields on a voxel-by-voxel basis using Spearman correlations. Rank correlations were applied given non-normality of E-field vector magnitudes. Each voxel was then color-coded by the resulting R value. Intuitively, this voxel-wise value expresses how the degree of stimulation correlated with memory improvement, across the group. This was done for both tasks separately and across the two tasks. Visualization of sweetspots in 2D was performed using 3D Slicer (https://www.slicer.org/^91^). Stimulation of areas with higher R values should be associated with greater memory benefit and to statistically validate this assumption, these sweetspots were cross-validated across the two tasks, by calculating the sweetspot exclusively on data from one experiment to predict the ranks of memory improvement of patients in the other sample, and *vice versa*. To do so, a sweetspot was calculated for one memory task first. For each patient of the other memory task, a sweetspot score was calculated by multiplying the R-values of this sweetspot with the mean of E-field vector magnitudes intersecting with it, for each patient. These sweetspot scores were then correlated with empirical memory improvements of the other (unseen) task.

###### Probabilistic Tractography.

For patients OCD1–7, we also employed a separate pipeline for electrode localization and tractography in MRI native space. Brain imaging data were analysed using FSL 5.0.6 (http://fsl.fmrib.ox.ac.uk/fsl/fslwiki/). Non-brain tissue was removed from the pre-operative T1-weighted images using FSL-BET. Skull stripped post-operative CT images were co-registered to the pre-operative T1 image using FSL-FLIRT affine linear transformation. Post-operative CT images were again thresholded at an intensity of 1500 Hounsfield units to retain just the electrode artefacts in the same orientation as the T1 image. The positions of the extreme of the lowest/highest tip of the most ventral/dorsal contacts were visually identified. In one case it was necessary to overlay a post-operative T1-weighted image for a correct estimation of the contact’s position. We computed the coordinates of the centroid of the contacts using trigonometric functions. A volume of activated tissue (VAT) was defined by linearly scaling up ellipsoids centered in the contact centroids according to a DBS spatial activation spread model^92^. The original size of the ellipsoids was *a*=1.93mm; *c*=1.50mm for the deepest NAc electrode, and *a*=1.63mm; *c*=1.20mm for the second NAc electrodes, where *a* is the perpendicular radius to the electrode, and *c* is the transversal radius. Our choice of volume was extrapolated (approximately) from previous characterization of the spatial extent of axonal activation during bipolar stimulation using artificial neural networks (based on Medtronic 3389 DBS electrode)^92^.

DWI data were pre-processed using FSL-BET for non-brain tissue removal and FSL-FDT for eddy currents correction. Estimation of the diffusion parameters was performed following a Bayesian approach^93^, using a multi-shell model for the fitting of the parameters^94^. White matter connectivity was quantified using probabilistic tractography with FSL-Probtrackx (http://fsl.fmrib.ox.ac.uk/fsl/fsl-4.1.9/fdt/fdt_probtrackx.html). Three tractography analyses were performed: 1. Using the bipolar NAc VAT masks for both left and right hemispheres as seeds and the hippocampal ROIs as waypoint and termination targets: 2. A two-step reconstruction using first the bipolar NAc VAT masks as seed and a midbrain mask^95^ as target, and second the midbrain mask as seed and the hippocampal ROIs as waypoint and termination target. Patient-specific hippocampus, caudate and NAc masks were extracted using Freesurfer 5.1.0.

Tractography parameters in the *probtrackx2* tool were five-thousand pathways per voxel in the seed ROIs, a maximum length of 2000 steps and a step-length of 0.5mm. Pathways with steps in which a sharp angle of 60° or higher occurred were discarded. The tractography maps were transformed to probabilities, dividing by the total number of pathways in the map. Then the maps were binarized by thresholding at a probability of 0.001 for the NAc VAT-hippocampal tractography and greater than 0 for the two-step NAc VAT-VTA-hippocampal reconstruction. In the two-steps reconstruction, the two probability maps were averaged before thresholding.

The *b*0 images were co-registered to the pre-operative T1 image using FSL-FLIRT affine linear transformation and later normalized to the 1mm^3^ T1 MNI template, using non-linear transformations from FSL-FNIRT. These transformations were concatenated and applied to the thresholded tractography maps. Then the two-steps tractography maps were summed across patients in order to represent the frequency in which a voxel within a VTA mask^95^ is reached across the group of 7 patients.

For the representation of the connectivity between the VAT and the hippocampus we used OpenWalnut software with the tool for visualization of boundary surfaces.

#### Exp 3: Sweetspot stimulation study.

This experiment followed the same protocol as Exp 1, except that stimulation was delivered in periods 4 and 6 of the encoding task. In addition, the bipolar contact pairs selected for stimulation were those whose VATs were physically closest to the memory sweetspot defined above.

#### Exp 4: Rodent study.

##### Animals

Data from 3 male Sprague-Dawley rats (250–300 g) are reported. Animals were purchased from Janvier Labs (France) and maintained under a 12/12-h light/dark cycle (lights on 07:00–19:00 h) at room temperature (22±2 °C), with free access to food and water. Rats were housed in groups of five and adapted to these conditions for at least 1 week before experimental manipulation. All experiments were approved by the local authorities (IN-CSIC) and were performed in accordance with Spanish (law 32/2007) and European regulations (EU directive 86/609, EU decree 2001–486).

##### Rat Neurosurgical procedure

All experiments were performed under urethane anesthesia (1.3 g/kg, i.p.). Stimulating electrodes consisted of glass-coated carbon fiber bipolar electrodes to minimize artifacts in the MR images as shown before^96^. Stimulating electrodes were implanted using standard surgical and stereotaxic procedures^50, 51, 97^ to target the shell of the Nucleus Accumbens (from bregma: 1.9 mm anterior, 0.8 mm lateral and 6.5 mm ventral to the dural surface)^98^. The final position of the bipolar carbon electrode was verified using high resolution anatomical (T2-weighted) MR-images.

##### Rat Deep-Brain Stimulation protocol

Charge balanced, left unilateral, bipolar stimulation at 150 μA (3.5 V) was delivered at 130 Hz (pulse width 60 μs) using a constant current source and a pulse generator (STG2004, Multichannel Systems, Reutlingen, Germany). DBS was applied in a block design (4 s ON, 26 s OFF) repeated 10 times per trial, with trials repeated 5 times per subject.

##### MRI Experiments and Data Analysis

The MRI experiments were carried out in a horizontal 7 Tesla scanner with a 30 cm diameter bore (Biospec 70/30, Bruker Medical, Ettlingen, Germany). The previously prepared urethane-anesthetized animals were placed in a custom-made animal holder with adjustable bite and ear bars, and positioned on the magnet bed. The animals were constantly supplied with 0.8 l/m O2 with a face mask and temperature kept between 37.0–37.5 °C through a water heat-pad. The temperature, heart rate, SpO2 and breathing rate were monitored throughout the session (MouseOx, Starr Life Sciences, Oakmont, US).

MRI acquisition was performed in 15 coronal slices using a GE-EPI sequence applying the following parameters: FOV, 25 × 25 mm; slice thickness, 1 mm; matrix, 96 × 96; segments, 1; FA, 60°; TE, 15 ms; TR, 2000 ms. T2 weighted anatomical images were collected using a rapid acquisition relaxation enhanced sequence (RARE): FOV, 25 × 25 mm; 15 slices; slice thickness, 1 mm; matrix, 192 × 192; TEeff, 56 ms; TR, 2 s; RARE factor, 8. A 1H rat brain receive-only phase array coil with integrated combiner and preamplifier, and no tune/no match, was employed in combination with the actively detuned transmit-only resonator (Bruker BioSpin MRI GmbH, Germany).

Functional MRI data were analyzed offline using our own software developed in MATLAB, which included Statistical Parametric Mapping package (SPM8, www.fil.ion.ucl.ac.uk/spm) and FSL Software. After linear detrending, temporal (0.015–0.2 Hz) and spatial filtering (3 × 3 Gaussian kernel of 1.5 sigma) of voxel time series, a cross-correlation analysis was applied with a simple boxcar model shifted forward in time, typically by 2 s or a boxcar convolved with a gamma probability density function (HRF). The results were largely comparable with all methods tested. Functional maps were generated from voxels that had a significant (*P* < 0.001) component for the model and they were clustered together in space (cluster size = 14; calculated with Monte Carlo simulation).

Regions of interest (ROIs) extracted using a rat atlas registered to the functional images^99^ were used to compute the amplitude of the evoked BOLD signal responses (as a percentage relative to a pre-stimulus baseline of 6 s) and volume of brain tissue activated relative to the ROI (number of active voxels divided by the total number of voxels in the region).

## Figures and Tables

**Figure 1:**
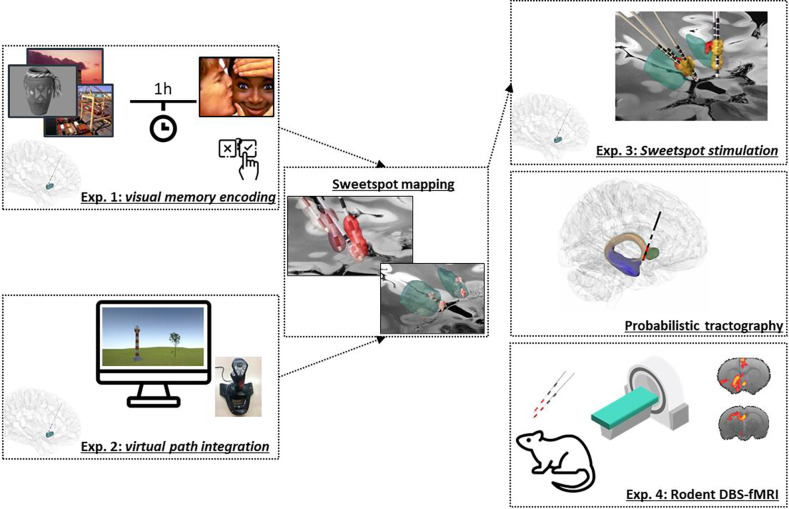
Experimental Outline. The effect of deep brain stimulation to the nucleus accumbens was investigated in three experiments. In a first study, we tested whether NAc-DBS during encoding enhances subsequent recollection of visual stimuli (Exp 1) in patients suffering from obsessive-compulsive disorder and one patient with anorexia nervosa. In a second study, we tested for NAc-DBS effects on spatial memory accuracy in a virtual navigation task (Exp 2). Next, we applied spatial mapping of DBS-induced memory enhancement across the two memory tasks to establish a stimulation “sweetspot” in the posterior dorsomedial NAc extending into the medial septum, associated with memory enhancement. In a subsequent 3 patients, the task from Exp 1 was repeated, stimulating the electrode contacts nearest to the sweetspot (Exp 3). Analysis of patient pre-operative diffusion weighted images revealed that structural connectivity places the stimulation site within a hippocampal-VTA circuit. In a fourth experiment, we acquired whole-brain functional MRI in rats to assess local and distant effects of NAc-DBS on neuronal activity (Exp 4).

**Table 1.** Patient details.

Patient	1	2	3	4	5	6	7	8	9	10	11	12	13	14	AN1	15	16	17
**Hospital**	HCSC	HCSC	HCSC	HCSC	HCSC	HCSC	HCSC	HUP	HCSC	HCSC	HUSE	HUP	HUP	HUP	HCSC	HUP	HUP	HUP
**Gender**	f	f	f	m	m	m	m	m	m	f	m	m	m	f	f	f	m	f
**Age at surgery**	49	37	28	28	50	36	20	31	21	34	22	38	26	34	37	55	30	41
**Age at Onset**	9	9	7	10	9	8	8	23	NA	NA	11	18	11	22	9	25	5	22
**Handedness**	R	R	R	R-L	R	R	R	R	NA	NA	NA	NA	NA	NA	R	R	R	R
**Schooling (years)**	15	8	15	11	9	12	10	NA	NA	NA	NA	NA	NA	NA	18	17	12	12
**Diagnosis**	OCD	OCD, MDD	OCD	Axis I: OCD Axis II: SPD	OCD	OCD	OCD	OCD	OCD	OCD	OCD	OCD	OCD	OCD	AN	OCD	OCD	OCD
**DSM-IV-TR codes**	300.3	300.3/296.3	300.3	300.3	300.3/305	300.3/303.9/292/300.21	300.3	NA	NA	NA	NA	NA	NA	NA	307.1/296.3	300.3	300.3	300.3
**Comorbidity**	None	Unspecific recurrent depressive disorder	None	Schizotypal personality disorder	Alcohol abuse	Alcoholism/Other recreational drug dependence/Social phobia	None	None	NA	NA	NA	NA	epilepsy	NA	Depressive disorder	None	None	None
**Obsessions**	Contamination/Tab oo thoughts (aggressive)	Symmetry/Contamination	Taboo thoughts (religious, aggressive)/Conta mination/Doubts	Symmetry/Ordering/Taboo thoughts, (aggressive,sexual)	Doubts,Contamination	Taboo thoughts (religious, aggressive)	Taboo thoughts (magic thinking)/Contamination	Contamination	NA	NA	NA	NA	NA	NA	NA	Contamination/Order	Hypochondriasis	Contamination
**Compulsions**	Washing	Ordering/Symmetry/Washing	Cleaning/Checking	Ordering, symmetry	Checking /Cleaning	Avoiding behaviors /Checking	Ordering	Washing/Repetition	NA	NA	NA	NA	NA	NA	NA	Washing	Checking	Washing
**Drug therapy (mg/day)**	Aripiprazole/Clonazepam/Valproic acid/Venlafaxine	Clomipramine/Sertraline/Flurazepam	Sertraline	Clomipramine/Sertraline/Oxitriptan	Clomipramine/Diazepam	Venlafaxine/Quetia pine	Clomipramine/Sertraline/Olanzapine/Lorazepam	Fluvoxamine/Clomipramine/Olanzapine/Clonazepam	Oxitriptan/Escitalopram	NA	NA	Fluvoxamine/Quetiapine/Levetiracetam/Biperiden	Amisulpride/Sertraline/Clotiapine/Clonazepam/Haloperidol/Lormetazepam/Lacosamide	Clorazepate/Fluvoxamine	Desvenlafaxine/Tianeptine/Lamotrigine/Clonazepam/Gabapenin/Quetiapine/Haloperidol	None	None	Sertraline
**Other prior therapies**	CBT/STPP/GPT	CBT	Nil	TMS	CBT	Nil	CBT	NA	NA	NA	NA	NA	NA	NA	CBT/GPT	Imipramine/Escitalopram/Clorimipramine	Risperidone/Clorazepate/Alprazolam	Quetiapine
**Preoperative YBOCS**	36	32	29	13^†^	38	36	40	34	24	NA	33	34	35	35	NA	35	35	33
**%YBOCS change(∅ contact 0&1)**	43	17	33	19	30	NA	13	NA	9	NA	39	NA	NA	26	NA	NA	NA	NA
**Best contact (R/L)**	2	0/2	2/3	1	3	3	NA	2	2	NA	NA	3	8/0	2	NA	NA	NA	NA
**% YBOCS change best contact**	97	25	45	23	47	63	NA	65	54	NA	NA	29	0	14	NA	NA	NA	NA
**Beck Depression Inventory**	27	43	24	22	37	51	NA†	NA	NA	NA	NA	NA	NA	NA	42	Hamilton 17	28	17
**STAI-S**	40	45	44	24	53	58	NA	NA	NA	NA	NA	NA	NA	NA	46	31	43	
**STAI-T**	49	42	45	40	48	60	NA	NA	NA	NA	NA	NA	NA	NA	52	50	49	
**Clinical Global Impression- severity scale**	6	6	6	6	6	6	7	NA	NA	NA	NA	NA	NA	NA	7	5	5	5
**Global Assessment of Functioning**	30	20	25	25	25	25	25	40	NA	NA	NA	NA	NA	NA	30	40	40	45
**Electrode model**	3391	3391	3391	3391	3391	3391	Boston	Boston	3391	3389	3391	3391	Boston	3391	3391	Boston	Boston	Boston(L) 3391 (R)
**Experiment performed**	1	1	1	1 & 2	1 & 2	1 & 2	1 & 2	1 & 2	2	2	2	2	2	2	1 & 2	3	3	3

Abbreviations, AN: anorexia nervosa; CBT: Cognitive Behavioral Therapy; DSM-IV-TR Diagnostic and Statistical Manual of Mental Disorders, 4th Edition, Text Revision; HCSC: Hospital Clinico San Carlos, Madrid; HUSE: Hospital Universitario Son Espases, Palma de Mallorca; HUP: Hospital Universitario de La Princesa, Madrid; MDD: Major Depression Disorder; NA: Not acquired; OCD: Obsessive-Compulsive Disorder; SPD: Schizoid Personality Disorder; STAI-S: State-Trait Anxiety Inventory-State version; STAI-T: State-Trait Anxiety Inventory-Trait version; STPP: Short-Term Psychodynamic Psychotherapy; TMS: Transcranial Magnetic Stimulation.

* At disease onset, this patient presented with cleaning, checking and order compulsions, which responded to pharmacotherapy. His score on the Yale-Brown Obsessive Compulsive Scale is in the mild range, which reflects the relative insensitivity of this scale to primarily obsessive symptomatology.

† This patient scored 30 and 31 on the Hamilton Anxiety Rating Scale (HARS) and the Hamilton Depression Rating Scale (HDRS), respectively.

## Data Availability

The behavioral data and Matlab analysis scripts that support the findings of this study are available via OSF (https://osf.io/cjdeh/) with the identifier DOI 10.17605/OSF.IO/CJDEH. The DBS MRI datasets generated and analyzed during the current study are not publicly available due to data privacy regulations of patient data but are available from the corresponding author on reasonable request. A mask of the memory sweetspot resulting from our analyses, as well as the Lead Group file containing electrode coordinates is available (https://osf.io/cjdeh/). Supplementary Tables 4–13 contain raw behavioral data of Exp 1 and 2 and Supplementary Figure S6 provides absolute intersections between patients’ VAT e-fields and surrounding anatomical structures. Further data that support the findings of this study are available from the corresponding author upon reasonable request.
